# Comprehensive Research and Analysis of a Coated Machining Tool with a New TiAlN Composite Microlayer Using Magnetron Sputtering

**DOI:** 10.3390/ma14133633

**Published:** 2021-06-29

**Authors:** Štefan Michna, Iryna Hren, Jan Novotný, Lenka Michnová, Václav Švorčík

**Affiliations:** 1 Faculty of Mechanical Engineering, Jan Evangelista Purkyne University, Pasteurova 3334/7, 400 01 Usti nad Labem, Czech Republic; stefan.michna@ujep.cz (Š.M.); jan.novotny@ujep.cz (J.N.); lenka.michnova@ujep.cz (L.M.); 2 Deparment of Solid State Engineering, University of Chemistry and Technology Prague, Technická 5, 166 28 Praha 6, Czech Republic; vaclav.svorcik@vscht.cz

**Keywords:** magnetron sputtering, HIPIMs method, TiAlN layer, XRD analysis, EDS analysis, surface morphology, coating thickness, AFM microscopy

## Abstract

The application of thin monolayers helps to increase the endurance of a cutting tool during the drilling process. One such trendy coating is TiAlN, which guarantees high wear resistance and helps to “smooth out” surface defects. For this reason, a new type of weak TiAlN microlayer with a new composition has been developed and applied using the HIPIMs magnetron sputtering method. The aim of this study was to analyze surface-applied micro coatings, including chemical composition (EDX) and microstructure in the area of the coatings. Microstructural characterization and visualization of the surface structures of the TiAlN layer were performed using atomic force microscopy. To study the surface layer of the coatings, metallographic cross-sectional samples were prepared and monitored using light and electron microscopy methods. The microhardness of the test layer was also determined. Analyses have shown that a 2-to-4-micron thick monolayer has a microhardness of about 2500 HV, which can help increase the life of cutting tools.

## 1. Introduction

The HIPIMS method (high power impulse magnetron sputtering) is a newly developing special PVD coating technology. It features a very high power density (1000 W/cm^2^) per sputtering electrode, with very short selectable pulse times (50–200 μs), which produce a higher plasma density than standard magnetron sputtering (>10^19^ m^3^). HIPIMS is a progressive method of applying layers with a controlled flow of material on the substrate due to ionization of the sprayed material. The spraying of the coatings is carried out using a source of particles (targets) with kinetic energy, and suitable examples include, Ar + ions, and, for layer formation, also nitrogen, carbon, oxygen, etc. This technology is used mainly for dedusting various conductive and non-conductive elements (Ti, Al, Zr, C, Cu, B, Si, etc.) in various proportions and together with supplied gases (nitrogen, oxygen, methane, hydrogen, etc.) a combination of different coatings can be produced, including non-conductive or composite layers. The elements that need to be released for coatings are dusted off from the solid state to the gaseous state through targeted bombardment of the source by argon and krypton atoms. The released atoms are subsequently ionized and, together with the gas atoms that can be brought into the coating chamber, are directed from the source target to the surface of the instruments. The result of the composition of these atoms is a homogeneous, extremely smooth coating, without the presence of microcapsules. Despite its increased energy intensity, it is widely used in many areas of industry, such as aerospace, military, automotive, etc., to provide a high degree of wear resistance and to extend tool life.

The HIPIMS method was first described in Ehiasarian et al. [1] for the pre-treatment of substrates before the application of nitride coatings. In their experiments, the high content of metal ions in the bombardment flow promoted the etching of the substrate surface. During the experiments, it was found that this bombardment contributed to the formation of a metal-implanted region that maintained a strong bond between the coating and the substrate.

Another large-scale work was carried out by Tiron [2], which focused on improving the deposition rate parameter in HIPIMs technology by changing the magnetic field and the pulse duration configurations. During the experiments, ten different materials (C, Al, Ti, Mn, Ni, Cu, Zn, Mo, TA, and W) were used, which were subjected to magnetron sputtering and HIPIMs discharge, without and using an external magnetic field. The results showed that the use of an external magnetic field increased deposition rates by approximately 40% to 140%, for only the selected materials, namely Mn, Ni, Cu, and Zn. An advanced study was carried out by Santiago [3], which was aimed at the effect of positive pulses on HIPIMS deposition of hard carbon coatings. In the experiments, different amplitudes of positive voltage (100 to 500 V) were used when applying carbon DLC coatings. The results of the experiments showed that the best voltage used was 500 V, at which point an increase in the hardness of the carbon coating was observed from 9.6 GPA to 22.5 GPA. In their study, Sarakinos et al. [4] found that the rate of carbon deposition in HIPIMs is only about 1.3–1.5 times higher than in conventional DC deposition using the same cathode and the same average current. However, the films deposited with HIPIMs had a density of 2.27–2.67 g/cm^3^, measured by X-ray reflectivity [5]. It was found that the sp3 fraction of these films is from 20 to 40% when using a substrate bias UB = −125 to −175 V. On the other hand, there are no reports yet on the ratio of the carbon ion to the neutral carbon for HIPIMS carbon plasma. Only a few experiments have been carried out using HIPIMS for the deposition of carbon films [6,7,8], Cr [9,10], Nb [11], Al, Ti, W [12], and WC [13] to increase the adhesion of hard coatings, such as CrN or TiAlN [14]. On the other hand, the choice of metal for sputtering is usually made by taking into account the chemical affinity of the coating–substrate system [12]. As the main factors determining the level of adhesion, the chemical strength of the bond was identified together with the mechanical influence of the metal acting as a compliant interlayer reducing shear stress [14].

The HIPIMs method is a progressive method of layer deposition with control over the material flow to the substrate due to the ionization of the sprayed material. Therefore, extensive research and analyses of the TiAlN layer has been carried out. This layer was invented at the Faculty of Production Technologies of UJEP and has never been studied in a similar study. The aim of this work was to explore and analyze the TiAlN composite layer of a new composition. Improving the quality of the material and eliminating defects can be done by examining all factors affecting the HIPIMs process, such as, first of all, mapping the connectivity of the layer with the basic material. Increasing surface abrasion and wear resistance can be achieved by examining factors affecting the HIPIMS process, such as, in particular, mapping the thickness of the layer and its compactness on the surface of the product. The possibilities of using this alloy are extensive, from machine forging or cast aluminum alloys, milling steels, cast steels, and cast irons to machining refractory and Ti alloys.

## 2. Experimental Setup and Material

### 2.1. Experimental Material

**Figure 1:**
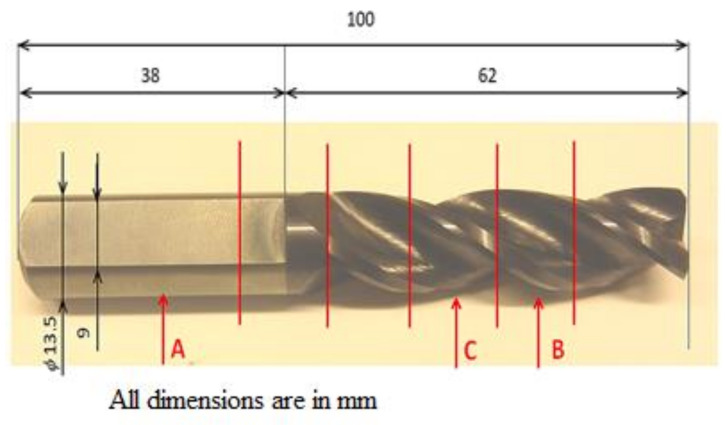
The whole body of the milling drill with a TiAlN coating applied to its working part with the determination of the places of selection for analyses (author source).

For the experiment, a cutting tool was used, which was based on tungsten carbide (WC) particles prepared using powder metallurgy and combined with Co (chemical composition: 88.6% W, 10.3% Co, 0.3% Cr, 0.05% Fe and 0.15% S). These materials exhibit a high hardness and their maximum application temperature is 800–900 °C. The sample was made in the form of a milling drill with the following parameters: length—100 mm and width—13.5 mm; one such sample is shown in Figure 1.

### 2.2. Experimental Setup

**Figure 2:**
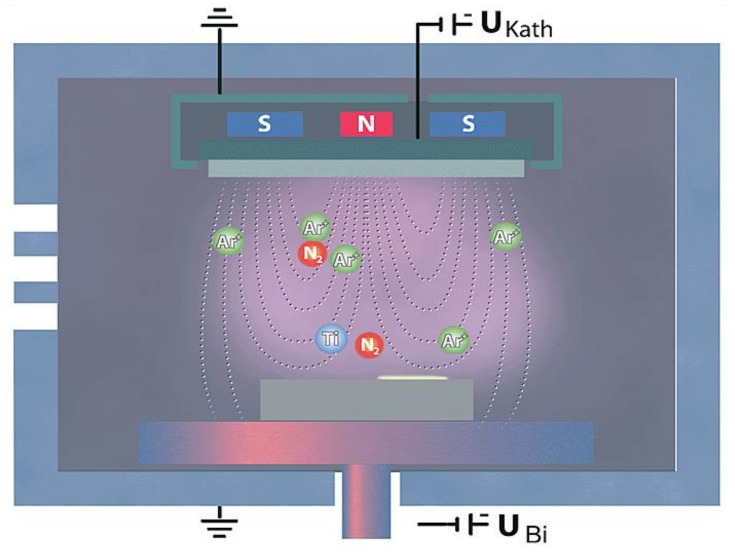
The principle of HIPIMs sputtering (source CemeCon) [18].

The principle of HIPIMS PVD sputtering is shown in Figure 2; the applied substance is the cathode (target) of the magnetron discharge. The discharge burns in a very dilute inert gas (usually Ar) in a vacuum chamber. Above a negatively charged target (500 to 1000 V), argon plasma is maintained, the positive ions of which are accelerated by the electric field to the target and, upon impact, sputter it. In front of the target, a magnetic field with a defined shape is created using an electromagnet or permanent magnets [15]. In this case, electrons that escape from the space in front of the target during classical sputtering must move along the helix, along the lines of force due to the Lorentz force [16,17]. Thus, their path in the vicinity of the target is significantly lengthened, the duration of their stay in the discharge region is also extended, and the probability of ionization of other atoms of the working gas increases. This makes it possible to maintain a discharge at a lower pressure (on the order of a tenth of a Pa) and at a lower voltage (on the order of a hundred volts). In particular, the lower pressure is positively reflected in the greater purity of the formed layers [18].

**Figure 3:**
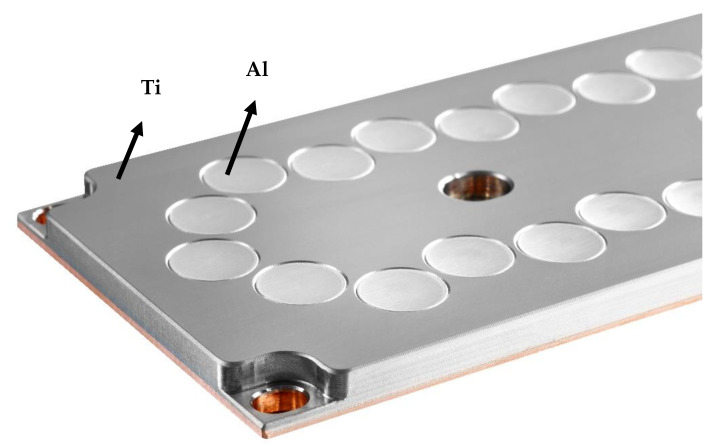
TiAl target with a nitrogen atmosphere for preparation of TiAlN coating (source CemeCon) [18].

The process for the preparation of our studied composite coating on the milling drill source targets that were used in the creation of the studied TiAlN coatings. These are plates in which bright colored aluminum rollers were pressed. In the coating process, the source target (Figure 3) was bombarded with argon and krypton atoms. The released Ti + Al particles, together with the supplied nitrogen gas, form a TiAlN coating on the surface of the milling drill. By changing the amount of Al rollers in the titanium plate, we were able to influence the resulting chemical composition of the coating, namely the ratio between titanium and aluminum.

Before the deposition processes, all samples were cleaned in an ultrasonic bath with ethanol for 10–15 min. After cleaning, the samples were placed in the chamber on a rotating table at an angle of 0°. The substrates were maintained at a constant temperature, determined before application to be 500 °C for 140 min. The TiAlN layer was applied using industrial coating device (CemeCon CC800/9XL HPPMS (CemeCon AG, Aachen, Germany)), in the HIPIMs mode. Pre-treatment with plasma etching was chosen to purify and activate the substrate surface prior to the deposition processes [19]. The spray device used had four cathodes capable of operating in the DCMS mode and two cathodes in the HIPIMS mode. The targets were installed on the HIPIMs cathode. Coatings were applied for 200 min using TiAlN targets at 5 kW, a frequency of 1000 Hz, and a pulse duration of 100 µs. To ensure uniformity of the composition, a substrate rotation speed of 10 RPM was used. After applying the TiAlN layers, the samples were cooled to 20 ± 5 °C for 30 min. A single layer of TiAlN was applied at a size of 3 ± 1 µm.

### 2.3. Experimental Methods

After plasma coating with a TiAlN layer, the structure and phase composition of the samples were analyzed using X-ray diffraction (XRD). The measurements were performed on a PANalytical X′Pert PRO X-ray diffractometer (PANalytical, Middle Watch Swavesey, Cambridge, UK) in a symmetrical Bragg–Brentano configuration under Cu_Kα_ radiation (λ = 1.5418 Å). The diffraction data were collected using an X′Celerator 1D detector in 2 theta range 20–80°. The concentrations of individual elements were analyzed using tube-above sequential wavelength dispersive X-ray fluorescence (WDXRF). The measurements were performed on a Rigaku ZSX Primus 4 device with a XMET 8000 Geo Expert handheld XRF analyzer (Oxford Instruments, Abingdon, UK) using the “soil” calibration. The data were evaluated using a non-standard fundamental parameters method, which allowed the determination of elements in the F-U range in concentrations from a hundredth of a ppm to 100%.

Five samples were prepared using conventional techniques—wet grinding and diamond emulsion polishing. All samples were manually prepared. The final mechanical chemical finishing was performed using a Struers OPS suspension (Struers, Ballerup, Denmark). After phosphoric acid etching, the structures of the material were observed and documented using light microscopy on a LEXT OLS 3100 confocal laser microscope from Olympus.

Scanning electron microscopy (SEM) was used for surface imaging of the coating with a SEM microscope (Tescan, Lyra3 GMU, Brno, Czech Republic) and for chemical analyses of the TiAlN micro-coating, EDS analyses were performed for the elemental distribution in the coating in terms of homogeneity and the structure of the coating. The accelerating voltage of the electrons was set to 5 kV. All the samples studied had to be dusted with metal to make the surface electrically continuous. The chemical composition and atomic abundances were studied using energy dispersive X-ray spectroscopy (EDS, X-Maxn analyzer, SDD detector 20 mm^2^, Oxford Instruments, Abingdon, UK). The acceleration voltage of the SEM-EDS method was set to 5 kV.

The study of surface morphology and the roughness of the tool samples was also assessed using atomic force microscopy (AFM) (Dimension ICON, Bruker Corp., Billerica, MA, USA). The QNM overhead mode in air was utilized for investigations. A silicon tip was used for the holder Si_3_N_4_, SCANASYST-AIR with a spring constant of 0.4 N·m^−1^. NanoScope analysis software was employed for data processing. The mean roughness value (Ra) represented the arithmetic mean of deviation from the median plane of the sample. The samples were analyzed in the Scan-Assyst mode (tapping mode) using a nitride lever (SCANASYST-AIR) with a Si tip. The typical tip radius of the curvature was smaller than 10 nm. The effective area represents the real area with the determination difference from the “basic” area in % (growth).

The microhardness test of milling the drill bit with the applied TiAlN coating was performed according to the CSN EN ISO 6507-1 standard on a microhardness tester (Mitutoyo HM-220) [20]. To measure the hardness, a 136° pyramidal diamond indenter creating a square indent was used. The nominal value of load was HV 0.2 (F = 1961 N, 200 g), which acted on the test specimen for 10 s. Due to the thickness of the layer, emphasis was placed on the chosen diagonal of the indenter in order to respect the ISO 6507-1 standard.

## 3. Results and Discussion

### 3.1. Analysis of the Chemical Composition of the Milling Drill

**Figure 4:**
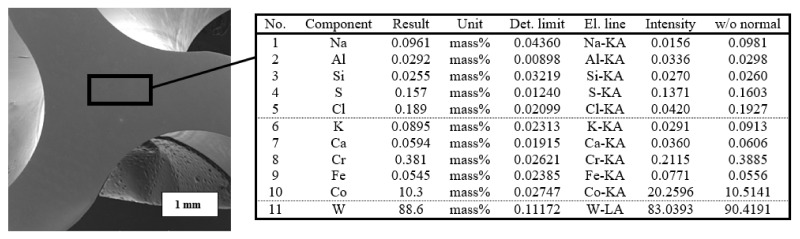
Area of the tool core examined by XRF and its elemental representation of the tool material.

Powerful milling drills, made of high-quality high-speed steel (HSS), which is ennobled with legures-tungsten, molybdenum, and chromium, are widely used in the automotive, aerospace, and military industries. Spring steel used in the production of industrial cobalt milling drills resists bending of the milling drill when drilling into a hard metal. The combination of tungsten and cobalt, together with the optimized geometry of the tip of the milling drill, keeps the working edges of the milling drill sharp four times longer. The prepared samples were examined using XRF analyses, where the area of the examined sample, including the result of the chemical composition, is shown in Figure 4. This analysis led to the exact composition of the body of the core of the milling drill before its subsequent coating. From the results of the XRF analyses, it is evident that tungsten with an 88.6% content is the constituent material of the milling drill and there is also about 10.3% cobalt serving as the bonding material.

**Figure 5:**
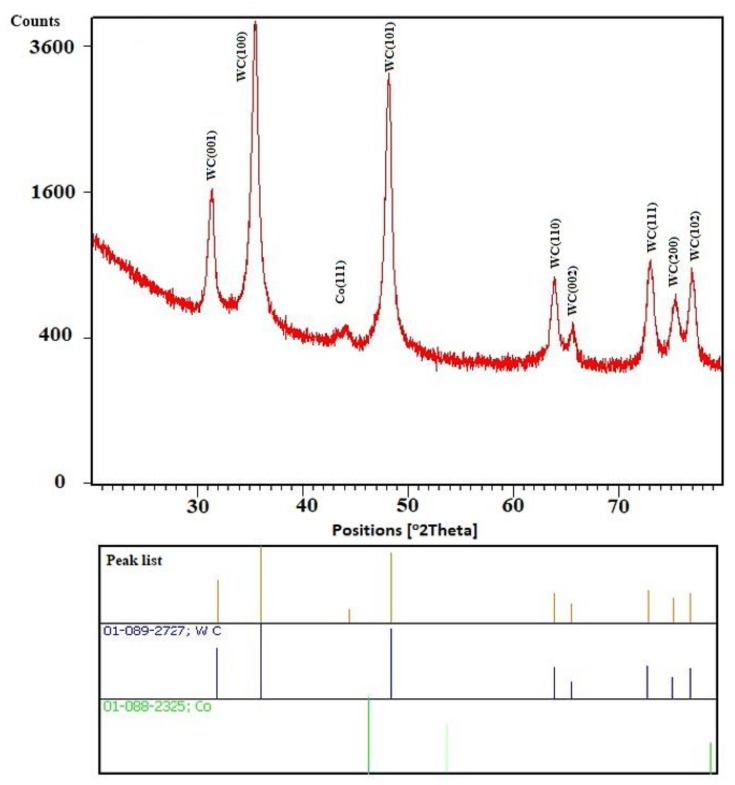
X-ray diffractograms of the tool core.

XRD phase analysis was performed to determine the phase composition of the tool. Figure 5 shows the obtained X-ray diffractograms with the diffraction peaks that were determined using a conventional θ-2θ X-ray diffraction scan of the studied tool. The diffractogram shows that the core of the milling drill is composed of WC. The spades belonging to the WC are wider, with a clearly larger FWHM (full width at half maximum) being the difference between the two values of the independent variable at which the dependent variable is equal to half of its maximum value. That is, the particles of the WC are fine-grained. Widespread at all peaks, WC (001), (100), (101), (110), (002), (111), (200), and (102) were approximately the same, so they were probably regular crystallites. Another peak at Co (111), in the diffractogram, opposite to the content of the WC, indicates the presence of another phase. It is noticeable that this is a cubic alpha phase, which confirms the content of Co as a binder. Thus, the quantification of the elemental composition in the conclusion is supported only by the chemical composition and not by the phase analysis.

### 3.2. EDS Coating Analysis

**Figure 6:**
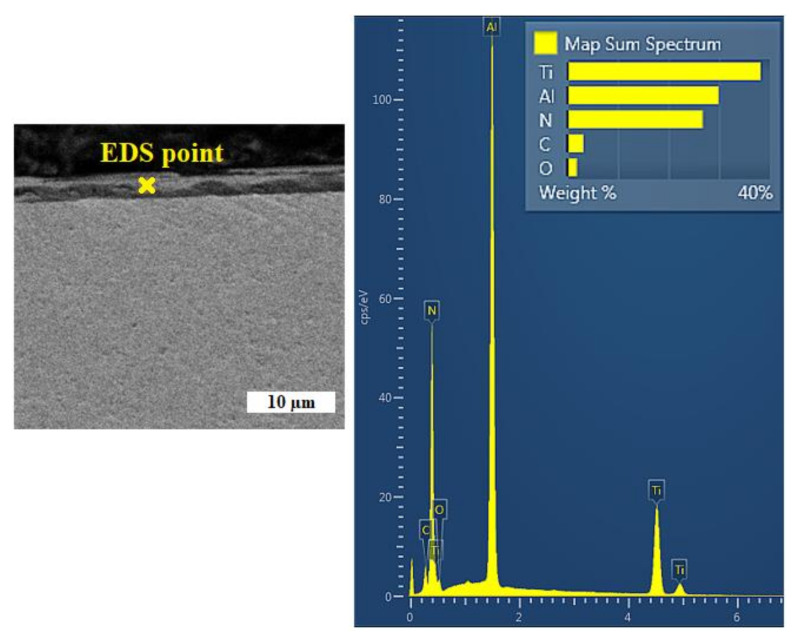
Selected site for spot analysis of the coating with the EDS recording of individual elements.

Among the current most-often created abrasion-resistant coatings can be found layers based on TiAlN. This layer was developed with the need to increase the oxidation resistance of tin layers. TiAlN layers compared to TiN, not only have higher oxidation resistance, but also a higher hardness of about 2000 HV, which allows the use of these coatings in intensive cutting conditions, such as high-speed machining and dry machining [21]. Point EDS analysis of the selected location (Figure 6) shows a composite TiAlN coating with the following percentage of elements: Ti-38.5%, Al-30.3%, N-27.3%, C-2.5%, O-1.5%. The analysis showed that the composite TiAlN coating contained titanium, aluminum and nitrogen. We performed a standard point analysis, followed by acquiring data from the elemental maps. Thus, the presented spectrum was acquired from multiple points on the sample, and is presented as a “combined” EDS spectrum, representing the average surface chemistry of the sample’s surface. The abundance of carbon in the elemental composition of the analyzed samples can be explained by the diffusion of this element from the substrate. Oxygen occurs in an air atmosphere and it is probably that during sputtering, oxygen atoms were entrained and their integration into the coating layer occurred. The ratio of titanium to aluminum can be adjusted by the aluminum content in the target [22]. In addition, carbon (2.5%) and oxygen (1.5%) were present in the coating in a small amount as polluting elements originating from the used technology, which Wagner also indicated in his study [23].

**Figure 7:**
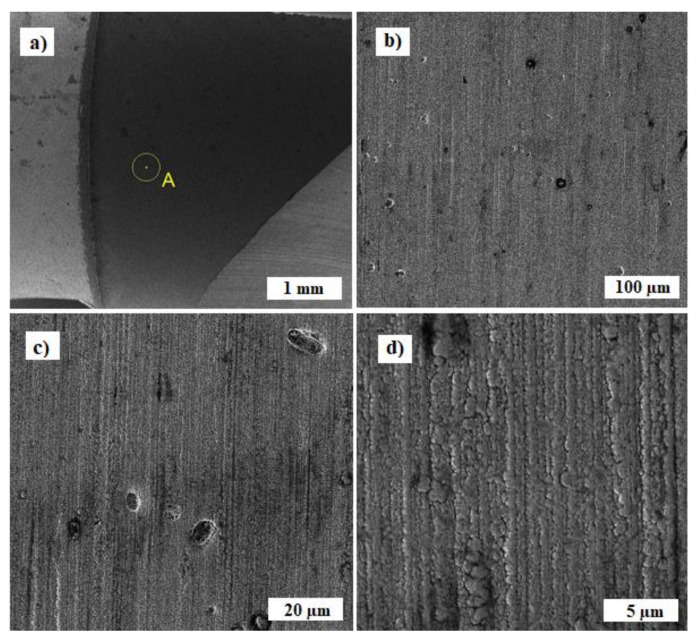
SEM secondary electron mode (SE) images of the place of examination, marked with the letter A (**a**), details (**b**–**d**) provide a detailed view of the morphology of the TiAlN coating.

The following images (Figure 7) show a coated layer of TiAlN, marked with the place of examination (letter A). There is a gradual approximation of the area shown in Figure 7a, so that the morphology of poles can be identified from the point of view of the applied technology of magnetron sputtering in the HIPIMs mode. In the TiAlN composite coating, isolated individual pores of max. 8–10 µm in size (Figure 7b) or separate foreign parts with a size max. of 10–15 µm (Figure 7c) are present. The images show that the morphology of the TiAlN coating was formed by lamination of the lamellas next to each other, where the individual lamellas are uniform over a narrow size range and reach a thickness in the range of 0.7–1.0 µm (Figure 7d). A similar morphology was studied in the publication by Wagner [23] or Jiang [24]. The same phenomenon was studied in all five samples examined in this study.

**Figure 8:**
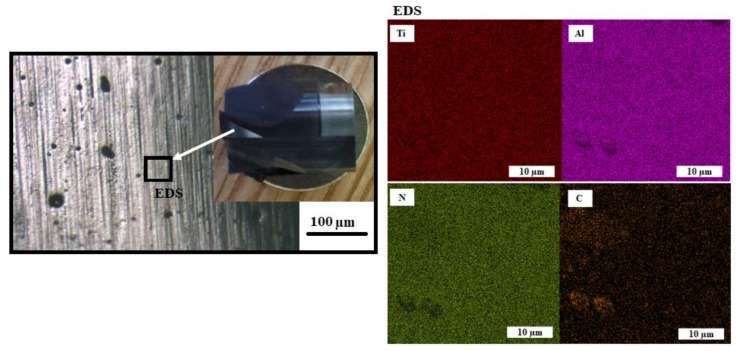
EDS analysis of the layout of individual elements in the TiAlN layer.

EDS analysis was also performed using a scanning electron microscope, which showed a very uniform distribution of individual elements in a given layer. The distribution of the elements used for coating (titanium, aluminum, and nitrogen), as well as carbon as an alloying element, was monitored and shown in Figure 8. From the point of view of the documented results of the distribution of the individual elements, it can be concluded that the distribution of all elements is very homogeneous, and two areas of greater inhomogeneity are noticeable for carbon compared to the rest of the studied area.

**Figure 9:**
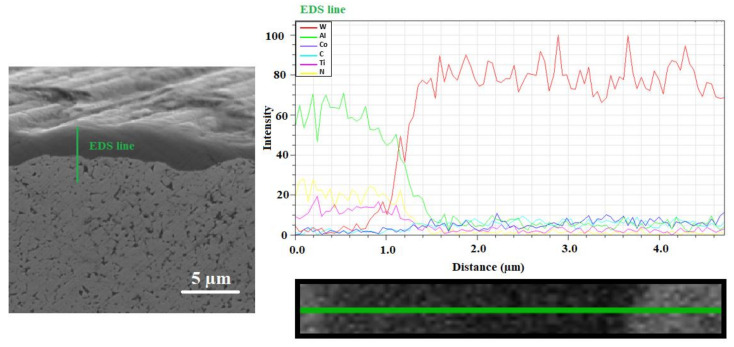
The morphology of the resulting micro-coat in a cross section (SEM) including the EDS measurement in a straight line.

SEM of the TiAlN coating are provided in Figure 9. A linear EDS analysis of the individual elements located in this area was performed.

The average thickness of the PVD coatings, as determined by SEM, was 1.5–1.7 µm (Figure 9). TiAlN coatings were characterized by a fine-grained microstructure with a low porosity, small internal stresses, excellent adhesion and a good thermal stability [25]. However, looking at the morphology of the micro-coating in detail, the compactness of this layer is observed to have a small number of pores. Moreover, in this area, with the help of linear EDS analysis, it can be seen that no expansion joint was found between the layer and the base substrate. Figure 9 also shows good adhesion between the layer and the base material. In the examined area of the layer (1–3 µm), a gradual decrease of Al is evident. This phenomenon was caused by the process of magnetron sputtering, in which Al was diffused into the base material [26]. A similar phenomenon can also be seen with nitrogen and very slightly with Ti (due to the fact that the differential potential of Ti compared to Al is much smaller [27]). During the magnetron sputtering process, W particles were also bombarded into the coating (at the bottom of the coating). This indicates a very good interconnection of the coating and the base material.

### 3.3. Analysis of the Surface of the Milling Drill by AFM Microscopy

**Figure 10:**
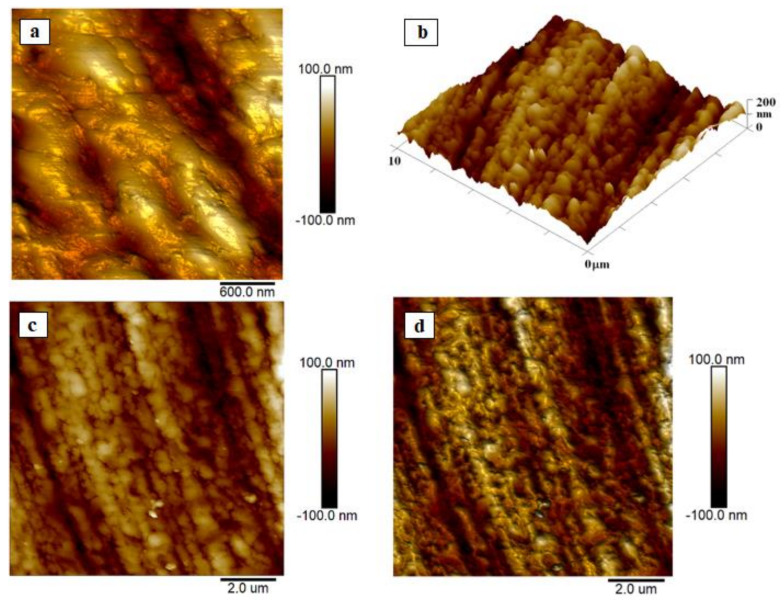
AFM analysis of the surface morphology and roughness: (**a**) analysis of tool and (**b**) 3D map of the tool-Ra = 20.5 nm, RMS = 25.8 nm, (**c**,**d**) detailed analysis of the tool-Ra = 17.5 nm, RMS = 22.4 nm.

Before carrying out the analyses, the samples were degreased with technical alcohol for 10 s. Subsequently, the surface morphology and roughness of the samples were studied using atomic force microscopy (AFM). As can be seen from the atomic force microscopy (and optical microscope) images, the surface of the milling drill also showed a sharper morphology (Figure 10) in accordance with the results from the scanning electron microscope. As introduced in [28], Ra and RMS values are dependent on the scan length; therefore, we introduced the saturated values in our paper.

Average roughness, *Ra*, is defined as the mean surface roughness representing the arithmetic mean of the deviations from the mean (zero) area. Compared to the interval of max/min heights (from −100 nm to 100 nm) it is commonly known that the value, as the result of *Ra* analysis, is much lower compared to *Z*min and *Z* max.

$$R_{a}=\frac{\sum_{i=1}^{N}\left|Z_{i}-Z_{cp}\right|}{N},$$

*Z_cp_* is the *Z* value of the center (zero) area, *Z_i_* is the current *Z* value, and *N* is the number of points in the scan (512 × 512 for our case).

The mean roughness (*R_a_*) is the mean of deviations from the mean plane of the sample. As can be seen from the images from the atomic force microscopy (and optical microscopy), the surface of the milling drill also has a sharper morphology (Figure 10a) in accordance with the results from the scanning electron microscope, with a roughness of 20.5 nm for a square of 10 × 10 µm^2^. In the analysis of the surface details (square 1 × 1 µm^2^), sharper nanostructures, homogenously distributed on the surface, with sizes in the range of 15–30 nm, are visible (Figure 10c,d). Shaded images (Figure 10a–d) were also used as alternative images for all 2D samples. The shaded versions and the corresponding “normal” 2D (Figure 10c,d) and 3D (Figure 10b) versions are one and the same, only “artificial lighting” from the software was used to make the details more visible. Unfortunately, no similar study of the roughness of these surfaces has been performed, so the results cannot be compared.

### 3.4. Analysis of the Connection of the TiAlN Coating on the Working Part of the Milling Drill

**Figure 11:**
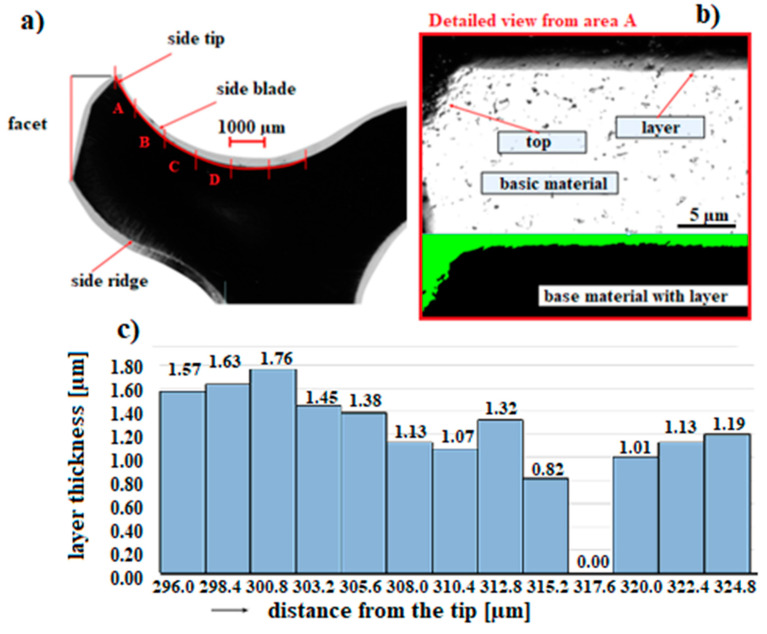
Display of the measured length of the coated TiAlN layer of the tool (**a**) showing the place of the coated layer (**b**) of the tool where the coating is almost completely absent (**c**).

Analysis and measurement of the thickness of the TiAlN coating was carried out in the working part of the milling drill, in a perpendicular section, using an OLS 3000 laser focal microscope (Figure 11a). The measurement was along the entire working length of the milling drill, in a perpendicular section from the tip, the side blade, and the side nail to the facet, in a total measured length of 6.4 mm.

**Figure 12:**
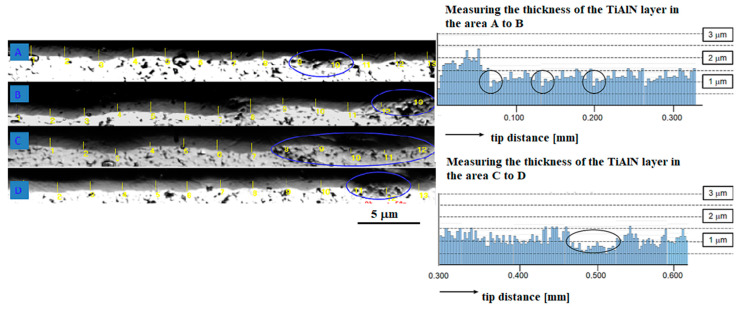
Representation of the thickness of the TiAlN layer of the tool in the measured section—indicating weak points A, B, C, D in the coated layer.

The measured thickness of the TiAlN layers range from 0.57 µm to max. 1.76 µm (Figure 11b), but, for example, about 0.3 and 0.5 µm from the tip, there are places that are practically without a layer (Figure 11c). From the overall visualization of the measured section of 6.4 mm, four places (marked with the letters A, B, C, D) are visible where the layer thickness is practically zero (marked with a circle in Figure 12). Therefore, it will be necessary to change the position of the milling drill a bit more when coating, so that even shielded areas are covered and a more even coating is achieved. Another method that could allow a uniform distribution of the coating is the combination of the TiAlN/TiAlCN coating mentioned by Tillmann [29] and Xiang [30].

### 3.5. Hardness Measurement

Table 1 shows the average value of the Vickers hardness measurements of the substrate and the measurements of coated layer hardness. Twenty measurements were done on each specimen and the average values were taken (10 for the basic substrate and 10 for the TiAlN coating). The determined hardness of the hard WC–Co metal was 1800 HV and corresponded to the value reported in the literature [31].

Due to the measured values of microhardness, one can clearly observe a steep increase in the values of microhardness of the applied TiAlN coating. The applied monolayer also showed high values of microhardness, max. 2500 HV. Similar results were achieved in the work of Wei [32]. In comparison with that publication, where the micro-layer of TiN/TiAlN had a microhardness of 3000 HV, the applied monolayer described in this article reached almost the same indicators, namely 2500 HV.

## 4. Conclusions

The analyses presented in this publication focused on the study of a new multi-layer micro-permeability for cutting tools from WC alloy, intended for drilling into hard metal. The following conclusions were drawn:

- From the results of XRF analyses, it is evident that tungsten with 88.6% content together with WC carbon was the constituent material of the milling drill;
- EDS analyses of the composite TiAlN coating showed that the morphology of the TiAlN coating was formed by lamination of the lamellas next to each other, where the individual lamellas are uniform over a narrow size range and reach a thickness in the range of 0.7–1.0 µm;
- EDS analyses proved very good homogenization of individual elements and their uniform representation in a given layer (titanium, aluminum, and nitrogen);
- Based on AFM analyses, we can conclude that the TiAlN layer showed sharper nanostructures, homogeneously distributed on the surface, with sizes in the range of 15–30 nm;
- The Ra parameter for the TiAlN layer was 17.5 to 20.5 nm;
- In some places, about 0.3 and 0.5 mm from the tip, there were places with practically no layer and, overall, in the 6.4 mm section, there are four places where the layer thickness was practically zero;
- The applied layer of TiAlN had a microhardness of about 2500 HV compared to the basic substrate, which had a microhardness of about 1800 HV.

From this it can be concluded that the technology of magnetron coating using the HIPIMs method will guarantee the required excellent homogeneity in the distribution of individual elements in the coating of the milling drill, but it will not guarantee a uniform thickness of the coating for more complex shapes. It will be necessary to change the position of the milling drill more when coating, so that even the shaded places are covered and a more even coating is obtained.

## Figures and Tables

**Table 1:** Representation of the hardness measurement result, HV.

Coating	Number of Layers	Hardness, HV
None	-	1800
TiAlN	Single layer	2500

## Data Availability

Data is contained within the article.
